# Musculoskeletal actinomycosis in children: a case report

**DOI:** 10.1186/s12879-021-06890-2

**Published:** 2021-12-07

**Authors:** Yani Mou, Qin Jiao, Yizhong Wang, Xiaolu Li, Yongmei Xiao, Lihua Zhao, Ting Zhang

**Affiliations:** 1 Institute of Pediatric Infection, Immunity and Critical Care Medicine, Shanghai Children’s Hospital, Shanghai Jiao Tong University School of Medicine, Shanghai, China; 2 Department of Orthopedics, Shanghai Children’s Hospital, Shanghai Jiao Tong University, Shanghai, China; 3 Department of Gastroenterology, Hepatology and Nutrition, Shanghai Children’s Hospital, Shanghai Jiao Tong University, Shanghai, China

**Keywords:** Actinomycosis, *Actinomyces*, Children, Ampicillin

## Abstract

**Background:**

Actinomycosis is a rare infectious disease caused by *Actinomyces*, especially in children. Here, we present a case of musculoskeletal actinomycosis in a 5-year-old girl from China.

**Case presentation:**

A 5-year-old girl presented with recurrent episodes of fever, pain, erythema, swelling, and festering sores on the right lower extremity, and pus was discharged from a sinus in the right foot. Magnetic resonance imaging (MRI) suggested subcutaneous soft tissue infection and osteomyelitis of the right crus. A bacterial culture of pus extracted from a festering sore on the right popliteal fossa detected the growth of *Actinomycetes europaeus*. The patient was cured with 7 weeks of treatment with intravenous ampicillin-sulbactam, followed by 6 weeks of treatment with oral amoxicillin-clavulanate with surgical debridement and drainage. There were no symptoms of recurrence during the 15-month period of follow-up.

**Conclusions:**

Pediatric actinomycosis is a rare and challenging infectious disease. Early accurate diagnosis and optimal surgical debridement are important for the management of pediatric actinomycosis.

## Background

Actinomycosis is a rare chronic, suppurative and granulomatous infectious disease caused by *Actinomyces* [1]. *Actinomyces* spp. are gram-positive and facultative anaerobic bacteria that usually inhabit the mucosa of the human oropharynx, gastrointestinal tract, urogenital tract, and female genital tract [1]. Oral colonization with *Actinomyces* is observed in the majority of children under the age of 2 years [2]. Actinomycosis usually occurs by contiguous growth of *Actinomyces* spp. through anatomic barriers where the mucosal barrier is disrupted. The most common species of human actinomycosis is *Actinomycetes israelii* [1, 3]. Actinomycosis affects all tissues and organs, such as orocervicofacial tissue, thoracic organs, abdominopelvic areas, central nervous system tissue, muscle and bone [4]. Actinomycosis has been described in all age and sex groups, with the highest incidence in middle-aged men [5]. The majority of reported pediatric actinomycosis occurs in the orocervicofacial areas and the abdomen [6–8]. Here, we report a case of musculoskeletal actinomycosis caused by *Actinomycetes europaeus* in a 5-year-old girl from China.

## Case presentation

A 5-year-old girl was admitted to our hospital due to a history of recurrent pain, erythema, swelling, festering sores on the right lower extremity, and discharged pus from a sinus in the right foot for 8 months. The routine antenatal ultrasound examination showed congenital absence of the right kidney, without a remarkable family history. The girl was born at full term with a low birth weight of 2000 g. She had nonsyndromic polydactyly in the right foot. A pustule on her right foot was observed after 3 days of birth, and her mother pricked it with a needle. A sinus was left after pus discharged from the wound for several days. At the age of 2 years, surgery was performed to remove the extra toes from the right foot at a local hospital.

The patient’s symptoms occurred 8 months prior to her admission to our hospital. She initially presented with pain, erythema, and swelling in the right popliteal fossa, accompanied by a high fever of 39 °C. Incision and drainage were performed at a local hospital, and then the symptoms improved. However, the patient’s symptoms reoccurred after 1 week of discharge, and drainage and intravenous cephalosporin were applied. Since then, the patient suffered recurrent episodes of pain, erythema, and swelling in her right popliteal fossa, which gradually extended to the right lower limb at 3 months prior to admission.

**Fig. 1:**
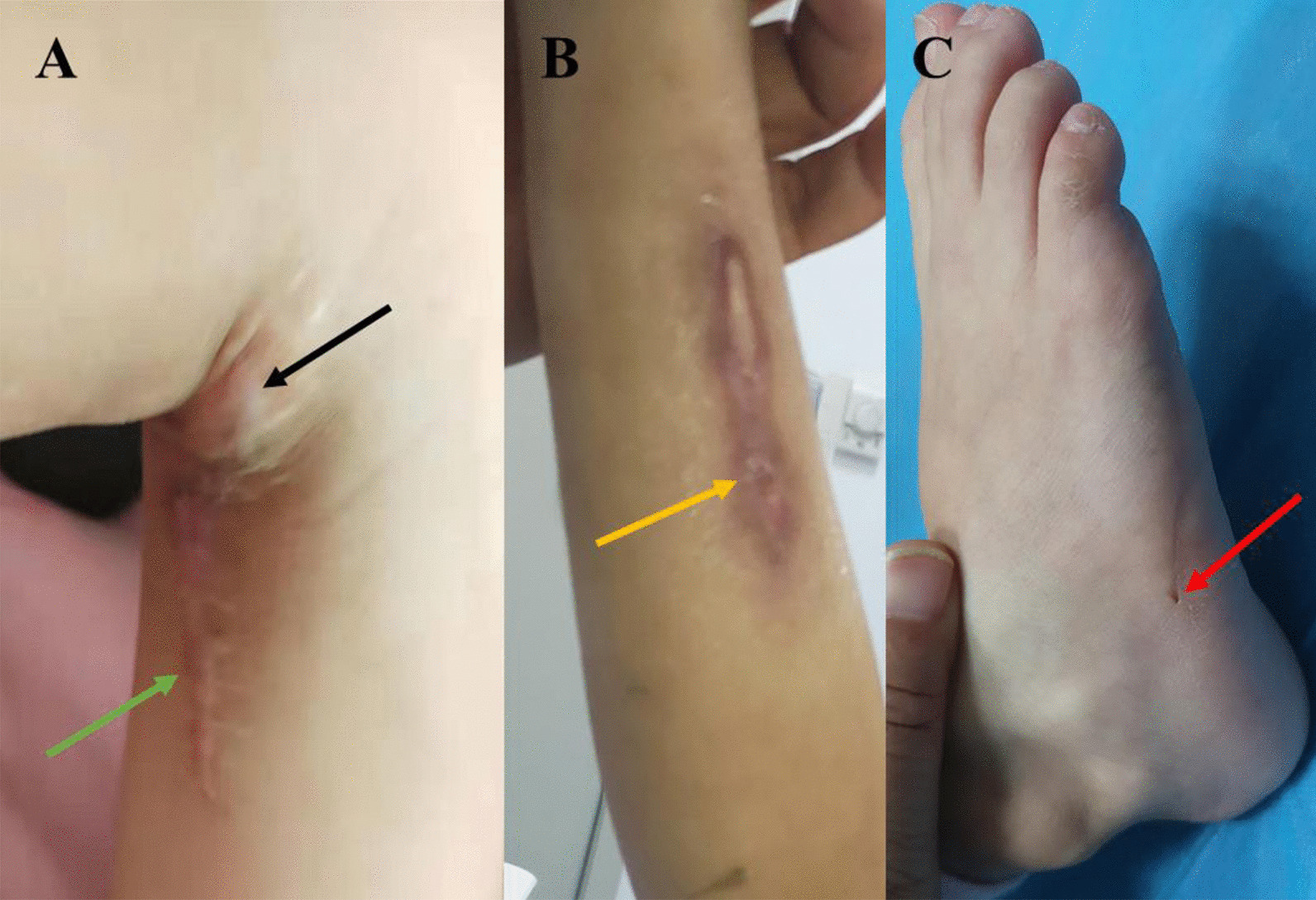
Injuries identified by physical examination. **A** The swollen area (black arrow) and a scar (green arrow) in the right popliteal fossa. **B** Two sinuses in the right crus (yellow arrow). **C** The sinus tract in the right foot (red arrow)

**Fig. 2:**
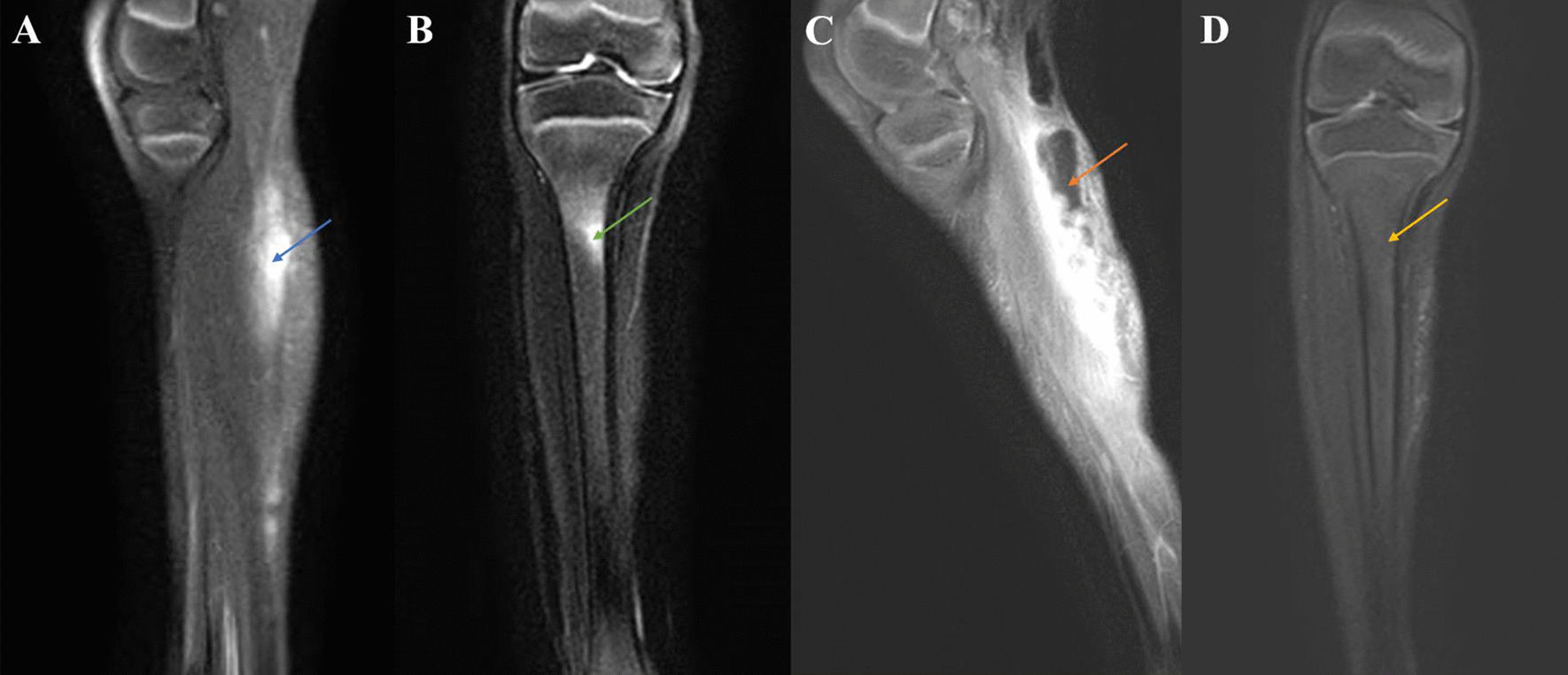
MRI findings of the right lower extremity. **A** Sagittal view of the right lower extremity MRI scan showing abnormal signal shadows (blue arrow) in the cutaneous and subcutaneous areas. **B** Coronal view of the right lower extremity MRI scan showing tibia bone marrow edema (green arrow). **C** Sagittal view of the right lower extremity MRI scan showing that the infection of the soft tissue had progressed, involving the right gastrocnemius muscle with pus cavity formation (orange arrow). **D** Coronal view of the right lower extremity MRI scan showing that the tibia bone marrow edema had been resorbed (yellow arrow)

**Fig. 3:**
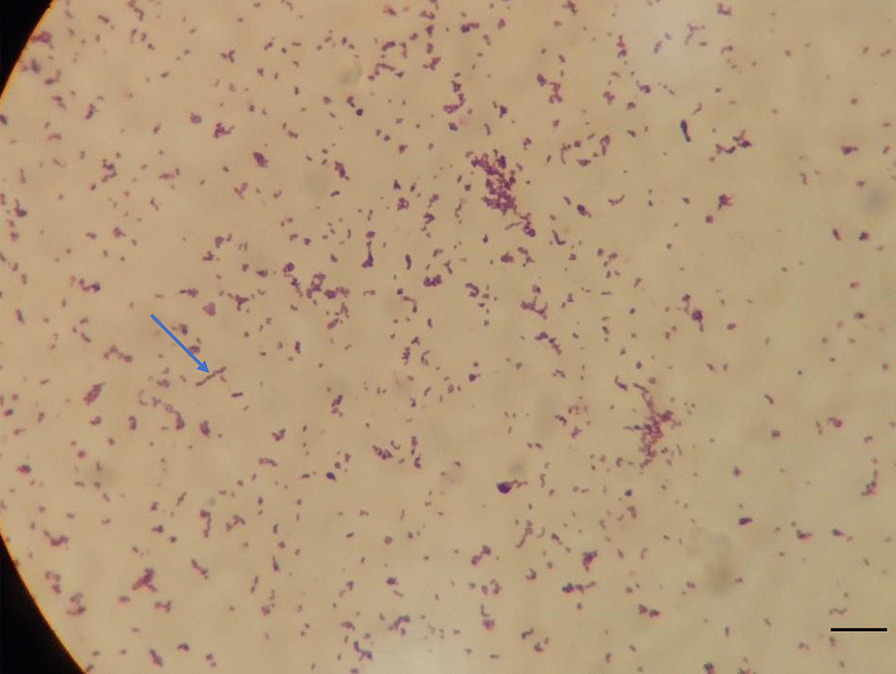
Bacterial culture of pus shows the growth of *Actinomyces*. Gram stain smear from a colony (100×, scale bar: 100 µM) showing gram-positive filamentous bacilli (blue arrow)

**Fig. 4:**
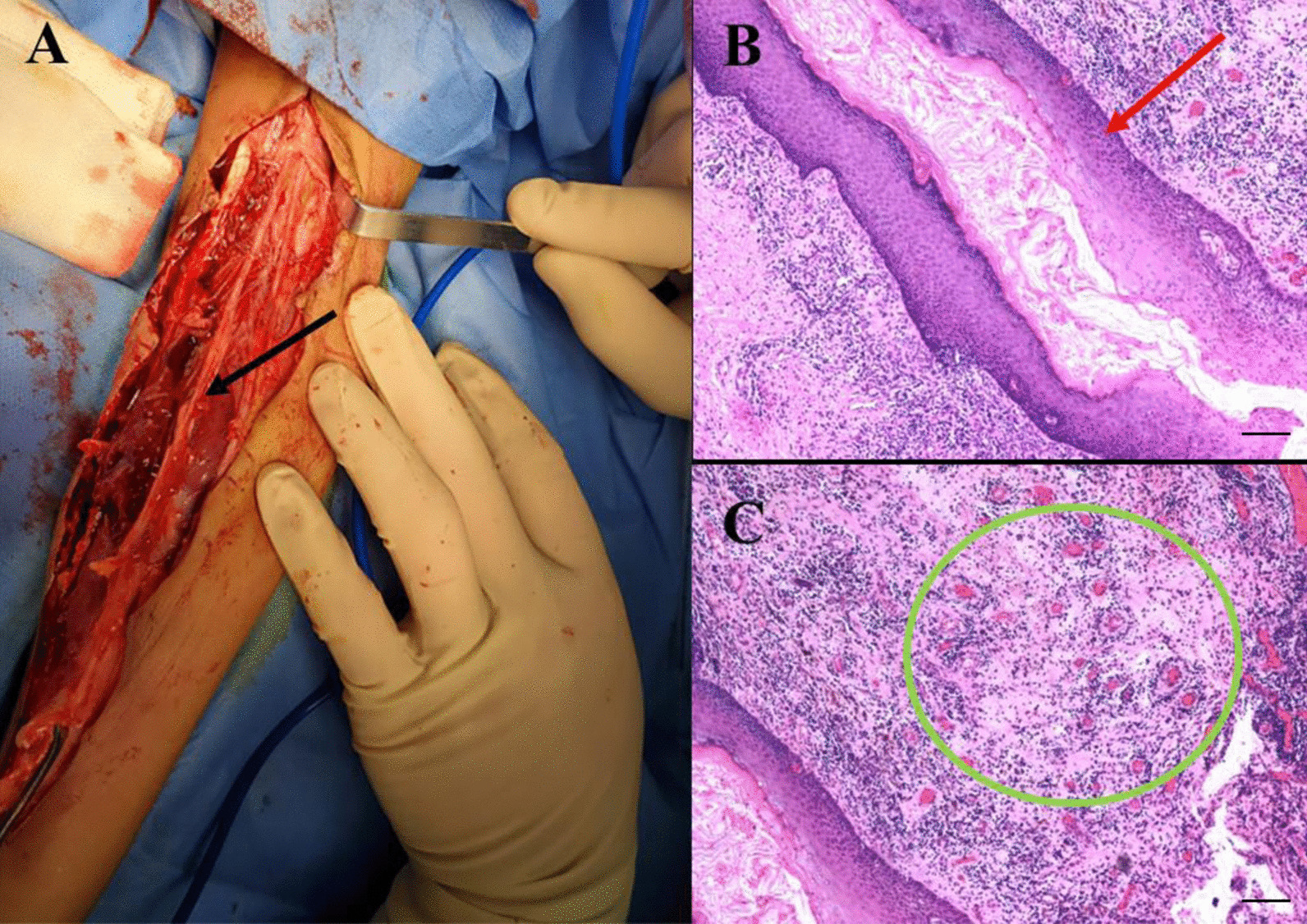
Surgery and histological evaluation of the surgical specimens. **A** Image showing debridement of the right lower extremity with a sinus tract (black arrow). **B** Pathological findings showing a lumen-like structure formed from the specimen of the sinus tract (red arrow, hematoxylin and eosin, 40×, scale bar: 250 µM). **C** Pathological findings showing granulomatous inflammatory lesions (green circle, hematoxylin and eosin, 40×, scale bar: 250 µM)

On admission, physical examination revealed that the patient’s liver and spleen were not palpable. The cardiovascular system, respiratory system and central nervous system showed no abnormalities. A scar of approximately 10 cm and a swollen area were found on the right popliteal fossa (Fig. 1A). Two sinuses on the right crus (Fig. 1B) and a draining sinus on the lateral side of the foot (Fig. 1C) were noted. Laboratory tests showed an elevated white blood cell count (WBC, 18.47 × 10^9^/L, reference range: 8–12 × 10^9^/L), C-reactive protein level (CRP, 108 mg/L, reference range: <5 mg/L), procalcitonin level (PCT, 0.15 ng/ml, reference range: <0.1 ng/mL), and erythrocyte sedimentation rate (ESR, 22 mm/h, reference range: 0–20 mm/h). The T-SPOT tuberculosis (TB) test, blood culture, bone marrow culture and smear were negative. Ova and parasite tests and parasite antigens were negative. Analyses of coagulation function and liver biochemical profiles were normal. Tumor markers (alpha-fetoprotein, carcinoembryonic antigen) and autoantibodies were negative. Lymphocyte subset analysis and immunoglobulin and neutrophil NADPH oxidase activity were normal. X-rays were unremarkable. Magnetic resonance imaging (MRI) revealed abnormal signal shadows in the inferior cortex, subcutaneous tissues of the popliteal fossa and behind the right crus (Fig. 2A, blue arrow) and tibiofibular bone marrow edema (Fig. 2B, green arrow), suggesting soft tissue infection and osteomyelitis. Bacterial culture of the pus extracted from the festering sore on the right popliteal fossa (blood AGAR, 37 °C, 72 h) detected the growth of *Actinomycetes europaeus*, which was confirmed by colony morphology, bacterial Gram stain smears (Fig. 3, ZEISS Primostar), and mass spectrometry. Therefore, the patient was diagnosed with musculoskeletal actinomycosis and was given intravenous ampicillin-sulbactam therapy. After 4 weeks of intravenous ampicillin-sulbactam (55 mg/kg, q8 hr) treatment, the patient’s symptoms persisted. Then, MRI was performed again, which showed that the bone marrow edema of the right tibiofibula had been resorbed (Fig. 2D, yellow arrow). However, the sagittal view of right lower extremity MRI scan showed the infection of the soft tissue had progressed, possibly involving the right gastrocnemius muscle with pus cavity formation (Fig. 2C, orange arrow). Considering the extensive lesions of the right crus and the presence of sinus tract in the patient, debridement was performed at the Department of Orthopedics. Large amounts of chronic inflammatory granulation tissue, disorganized fibrous connective tissue, and a sinus tract were observed (Fig. 4A). Irrigation was performed after removing the inflammatory granulation, fibrous connective tissue, and sinus tract, and a negative pressure drainage ball was placed. Pathological examination of a biopsy specimen from the sinus tract showed local fibrous hyperplasia with collagenization, endovillage squamous epithelium with hyperkeratosis, surrounding inflammatory cell infiltration, sinus tract formation (Fig. 4B, LEICA DM500), granulomatous inflammation with lymphoid hyperplasia, and hyperplasia with multinuclear giant cell reaction (Fig. 4C, LEICA DM500). The patient was discharged with significant symptom improvement after an additional 3 weeks of intravenous ampicillin-sulbactam (55 mg/kg, q8 h) treatment. Another 6 weeks of maintenance therapy with oral amoxicillin (32 mg/kg, q8 h) and clavulanate (9.2 mg/kg, q12 h) was given to the patient after discharge, and a favorable outcome was achieved.

The first follow-up was performed at the Department of Orthopedics after 2 weeks of discharge. The patient’s wound surface had healed, no articular contracture was found, and the MRI scan was unremarkable. The second follow-up was performed at the Department of Gastroenterology, Hepatology and Nutrition after 1 month of discharge. There were no fever, swelling, pain, or pus, and the routine blood tests were normal, including WBC count, ESR, and CRP levels. Then, the patient was followed up every month by phone. To date, there were no symptoms of recurrence, such as pain, erythema, or swelling, during the 15-month period of follow-up. Currently, monthly follow-up is ongoing to monitor the possible disease recurrence of the patient.

## Discussion and conclusions

Actinomycosis is an uncommon bacterial disease caused by *Actinomyces* infection. Normally, *Actinomyces* is a part of the human oral cavity flora, and the pathogenesis of actinomycosis involves a destruction of mucosal integrity followed by bacterial invasion [1]. Cervicofacial, abdominopelvic and pulmonothoracic sites are the most commonly affected locations of actinomycosis, and other clinical manifestations have been described, including in extrafacial bone and joints, the genitourinary tract, the digestive tract, and the central nervous system [1]. Pediatric actinomycosis is rare, accounting for a minority of all actinomycosis cases, and the most affected areas are cervicofacial and abdominopelvic locations [8, 9]. Although actinomycosis presenting as osteomyelitis in the pediatric population has been reported, most of the cases involved the mandible site [6]. In this report, we described a case of musculoskeletal actinomycosis in a 5-year-old girl. The patient suffered from recurrent episodes of fever, pain, erythema, and swelling in the right popliteal fossa for 8 months. MRI revealed soft tissue infection and osteomyelitis of the right crus, and the growth of *Actinomycetes europaeus* was detected by bacterial culture of the pus extracted from festering sores. Although the exact cause of bacterial infection is undetermined, it may be associated with the breach of skin barriers by sinus tract formation after birth and the surgical history of the right foot.

The diagnosis of actinomycosis is challenging, and it frequently mimics malignancy, TB, granulomatous disease, nocardiosis, or botryomycosis [1]. The gold standard for the diagnosis of actinomycosis is the isolation of *Actinomyces* spp. from tissue or pus from a usually sterile body site [10]. However, due to its anaerobic predilection and slow-growing nature, *Actinomyces* is difficult to culture [1]. A low sensitivity of cultures ranging between 20 and 50% for the diagnosis of cervicofacial actinomycosis was reported in previous studies [10, 11]. A literature review that analyzed 19 pediatric abdominal actinomycosis cases revealed a positive culture in 42% of the cases [8]. In addition to a positive culture, diagnosis is made from positive histopathology showing abundant granulation tissue, round granules with gram-positive bacteria, branching filaments oriented radially around the granules, or sulfur granules visible in drainage or pus [1]. Both a positive culture of *Actinomycetes europaeus* and a typical histopathology were observed in our reported case. In cases of actinomycotic osteomyelitis affecting extrafacial bone, the degree of progression, soft tissue reaction, existing sinus, and radiological findings should be considered [12]. Furthermore, molecular techniques, such as 16 S rRNA gene sequencing and mass spectrometry, can be used for the identification of *Actinomyces* [13].

Antibiotics are the first choice of actinomycosis treatment. *Actinomyces* is extremely susceptible to β-lactam antimicrobial agents, classically penicillin G or amoxicillin [1]. The recommended standard care of actinomycosis is high-dose intravenous penicillin G for 2–6 weeks followed by 6–12 months of prolonged oral penicillin or amoxicillin [1]. Most actinomycosis patients can be cured with antimicrobial therapy alone [14]. Surgical resection is indicated for voluminous abscess drainage, chronic sinus tract marsupialization, recalcitrant fibrotic lesion excision, or necrotic bone tissue debridement, as well as patients with poor antibiotic response [1]. Studies have shown that the antibiotic duration could be shortened for patients when they have undergone appropriate surgical resection of infected tissues [9, 15]. In our case, debridement was performed to remove chronic inflammatory granulation tissue, disorganized fibrous connective tissue, and the sinus tract. Then, the patient fully recovered after 3 weeks of intravenous ampicillin-sulbactam, followed by 6 weeks of maintenance therapy with oral amoxicillin and clavulanate. There was no recurrence of symptoms during the 15-month period of follow-up. Currently, monthly follow-up is ongoing to monitor the possible disease recurrence of the patient.

In summary, we reported a rare pediatric case of musculoskeletal actinomycosis affecting the right lower extremity. Multiple examinations are needed to make a differential diagnosis of actinomycosis, especially in children. Consistent with previous reports, our case showed that optimal surgical debridement, in addition to antimicrobial therapy, is effective in treating actinomycosis.

## Data Availability

All data generated or analyzed during this study are included in this published article.

## Abbreviations

CRP: C-reactive protein
ESR: Erythrocyte sedimentation rate
MRI: Magnetic resonance imaging
PCT: Procalcitonin
WBC: White blood cells
TB: Tuberculosis
